# MASH‐Ocean 1.0: Interactive platform for investigating microbial diversity, function, and biogeography with marine metagenomic data

**DOI:** 10.1002/imt2.201

**Published:** 2024-05-19

**Authors:** Yinzhao Wang, Liuyang Li, Qiang Li, Yaoxun Hu, Wenjie Li, Zhile Wu, Hungchia Huang, Zhenbo Lv, Wan Liu, Ruifang Cao, Guoping Zhao, Fengping Wang, Guoqing Zhang

**Affiliations:** ^1^ State Key Laboratory of Microbial Metabolism, School of Life Sciences and Biotechnology Shanghai Jiao Tong University Shanghai China; ^2^ National Genomics Data Center & Bio‐Med Big Data Center, CAS Key Laboratory of Computational Biology, Shanghai Institute of Nutrition and Health Chinese Academy of Sciences Shanghai China; ^3^ Shanghai Southgene Technology Co., Ltd. Shanghai China; ^4^ Hangzhou Institute for Advanced Study University of Chinese Academy of Sciences Hangzhou China; ^5^ State Key Laboratory of Genetic Engineering, Fudan Microbiome Center, School of Life Sciences Fudan University Shanghai China; ^6^ School of Oceanography Shanghai Jiao Tong University Shanghai China

## Abstract

A large number of oceanic metagenomic data and environmental metadata have been published. However, most studies focused on limited ecosystems using different analysis tools, making it challenging to integrate these data into robust results and comprehensive global understanding of marine microbiome. Here, we constructed a systematic and quantitative analysis platform, the Microbiome Atlas/Sino‐Hydrosphere for Ocean Ecosystem (MASH‐Ocean: https://www.biosino.org/mash-ocean/), by integrating global marine metagenomic data and a unified data processing flow. MASH‐Ocean 1.0 comprises 2147 metagenomic samples with five analysis modules: sample view, diversity, function, biogeography, and interaction network. This platform provides convenient and stable support for researchers in microbiology, environmental science, and biogeochemistry, to ensure the integration of omics data generated from hydrosphere ecosystems, to bridge the gap between elusive omics data and biological, ecological, and geological discovery, ultimately to foster the formation of a comprehensive atlas for aquatic environments.

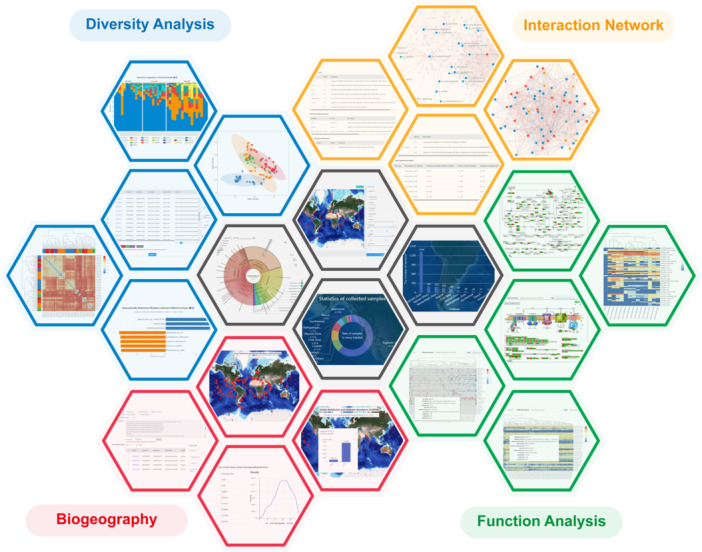

Oceans account for approximately 71% of the total surface area of Earth, making them the largest habitats for life on this planet. They contribute to over 90% of the total volume of the biosphere and are responsible for nearly half of the global total primary productivity [1]. It has been estimated that marine ecosystems host a remarkable number of over 10^29^ bacterial and archaeal cells [2]. These microbes exhibit vast diversity and are ubiquitously distributed across various oceanic environments, including both sea water and subsurface from intertidal zone, euphotic layer, and deep sea. Moreover, they thrive in special geological areas such as hydrothermal vents, cold seep regions, subduction zones, and oceanic crust. Active biogeochemical cycling mediated by these microorganisms through their carbon, nitrogen, and sulfur metabolisms can directly influence the Earth's climate [3]. Therefore, it is critical to understand the diversity, function, and biogeography of marine microbiome.

With the ongoing advancements in oceanic sampling technologies and high‐throughput sequencing platforms in recent years, scientists have discovered a wide variety of microorganisms with different ecological and geological functions in various marine habitats [4]. Microorganisms inhabiting distinct ecological niches may specifically play key roles in driving certain geochemical cycles and mediating the fluxes of carbon, nitrogen, and sulfur among sediment, sea water, and atmosphere [3]. Currently, an increasing number of metagenomic studies focusing on various types of oceanic environments have been reported [5]. For example, in sea water, archaea, bacteria, protist, and virus have been found to play significant roles in influencing the release of organic matter, mineralization, and fixation of carbon dioxide [6]. In sediments, the abundance and diversity of microorganisms is much higher than that in marine water bodies and primarily facilitates the anaerobic degradation of organic matter, sulfate reduction, methane production, and oxidation [7]. In summary, the analyses of multiple omics data have revealed a significant presence of microorganisms in oceanic environments that actively participate in the cycling of carbon, nitrogen, and sulfur elements.

Although high‐throughput metagenomic sequencing technology has greatly promoted microbiological research, each individual study has employed different analytical processes. These analysis results often show substantial heterogeneity when compared with each other. Only by accumulating and analyzing metagenomic data in a standard way, scientists would be able to reach robust conclusions and generate comparable results. Several platforms, such as Integrated Microbial Genomes and Microbiomes (IMG/M) [8] and Marine Metagenomics Portal (MMP) [9], support preliminary analysis of metagenomic or genomic data. Recent developments of online microbiome analysis platforms, for example, Wekemo Bioincloud [10] and MicrobiomeAnalyst [11], have also facilitated researchers in exploring the biological significance of metagenomic samples by providing multiple analysis tools and interactive visualization capabilities. Nevertheless, a gap still exists between elusive omics data and biological, ecological, and geological discovery. Here, we have established an online microbiome platform called the Microbiome Atlas/Sino‐Hydrosphere for Ocean Ecosystems (MASH‐Ocean, https://www.biosino.org/mash-ocean/), aiming to bridge this gap and achieve standardized, interactive analyses of massive metagenomic data, and to explore the knowledge atlas related to microbial mediated oceanic carbon, nitrogen, and sulfur cycling.

## RESULTS AND DISCUSSION

### Description of MASH‐Ocean 1.0

**Figure 1:**
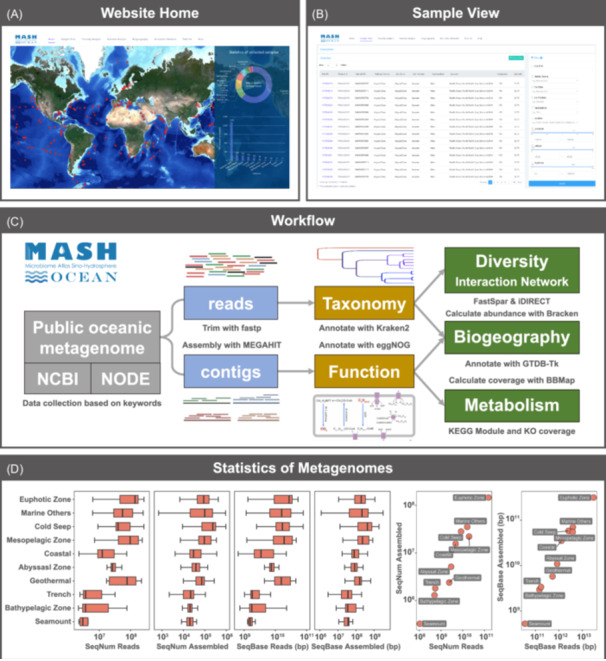
Overview of the MASH‐Ocean platform. (A) The front page; (B) sample display and selection panels; (C) the data analysis flow chart; (D) the statistics of metagenomes. The term “SeqNum_Reads” indicates the number of metagenomic clean reads, “SeqNum_Assembled” means the number of assembled contigs, “SeqBase_Reads (bp)” indicates the total sequence base of both metagenomic paired‐end reads, and “SeqBase_Assembled (bp)” means the sequence base of assembled contigs.

MASH‐Ocean is a website‐based platform (Figure 1A). This platform collects public metagenome data related to global marine environments and assembles and annotates these data with a uniform standard bioinformatic workflow. The current database contains annotations of microbial taxonomic features derived from metagenomic reads and metabolic functional features from assembled metagenomic contigs. These results can be visualized by online users without the well‐established knowledge of bioinformatics and omics. Three major advantages of the MASH‐Ocean platform are microbial diversity analysis, metabolic feature reconstruction and comparison using selectable metagenomic datasets globally, as well as interactive visualization of global distribution of the selected functional genes or microbial species (Movie S1).

### Public data used in MASH‐Ocean 1.0

All data within MASH‐Ocean 1.0 was collected from the National Center for Biotechnology Information (NCBI) Sequence Read Archive (SRA) (https://www.ncbi.nlm.nih.gov/sra) and the National Omics Data Encyclopedia (NODE) databases (https://www.biosino.org/node/) by September 2020 with a total of 75 keywords (Table S1). Metagenomes were also filtered out manually by their size (below 200 MB), descriptions such as “enriched,” “metatranscriptome,” and “amplicon,” as well as the restricted datasets from DOE Joint Genome Institute (JGI). In total, 2147 samples from different marine environments were selected for further analyses to build a basic knowledge pool for MASH‐Ocean 1.0 (Figure 1B). Additionally, MASH‐Ocean keeps downloading and filtering public oceanic metagenomic data to maintain and update this platform. The second batch of data (September 2023) has been downloaded and is under the analyzing process.

### Data process and data types

The MASH‐Ocean 1.0 workflow encompasses both reads‐level and contigs‐level analyses (Figure 1C,D). Specifically, the platform currently hosts two primary data categories: “clean reads” consisting of 210 billion sequences (totaling 44.2 trillion base pairs) and “contigs” comprising 257 million sequences (totaling 555.3 billion base pairs). For microbial taxonomy analysis, trimmed metagenomic reads were annotated by Kraken2 v2.1.3 [12], and the abundance was estimated by Bracken v2.6.1 [13]. For metabolic feature analysis, the trimmed reads were assembled by MEGAHIT v1.2.9 [14]. The contigs coverage was calculated by mapping the trimmed reads to each contig using BBMap v38.18 [15]. All contigs were subjected to open reading frame (ORF) prediction with Prodigal v2.6.3 [16]. The predicted ORFs were annotated by eggNOG database [17].

### Sample description, selection, and visualization

On this platform, all samples can be found in “Sample view” section on the website. The table here shows 10 key information of samples. Users can locate specific samples or narrow down the sample pool by providing key information in the navigation panel (Figure 1B). For the fields of Run/Project/Sample ID, we provide a field matching search mode, allowing users to match all complete fields and help them select the appropriate item by typing in part of the string. For the fields of Habitat source, Env Zone, Env Medium, Fraction, and Location, we provide a composite selection method that combines field matching with a drop‐down box to provide users with a convenient operation experience. Flexible selections are also supported by providing a drag bar with information of longitude, latitude, and water depth. The selected samples can be displayed on a global topographic map by clicking on the “Display Map” button, located at the top right of the sample table, to easily obtain a panoramic view of sample distribution.

### Diversity and ecological comparison analysis

**Figure 2:**
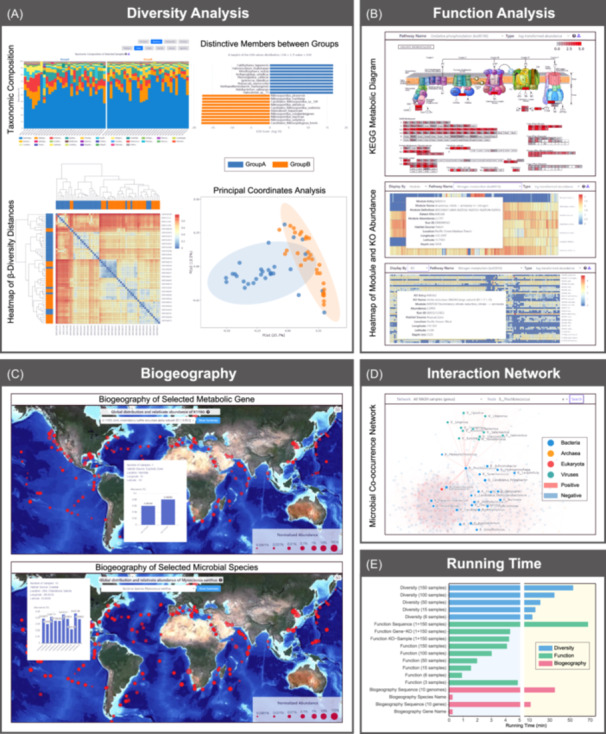
Analysis modules and running time of the MASH‐Ocean platform. (A) Diversity analysis module including taxonomic analysis, heatmap analysis, LEfSe analysis, and PCoA analysis of selected metagenomic samples using two groups (Group A and B) with 30 samples in each of the groups. (B) Comparison of metabolic functional genes from different metagenomic datasets. A Kyoto Encyclopedia of Genes and Genomes (KEGG) metabolic diagram is displayed when no more than three samples are selected. A heatmap is displayed when more than three samples are selected. (C) Biogeography of selected metabolic genes (e.g., *dsrA*) or microbial species (e.g., the species *Myxococcus xanthus*) in global metagenomic datasets. The size of the circle is proportional to the abundance of genes or species. A bar plot will show the relative abundance of the pointed metabolic genes or microbial species. (D) Microbial co‐occurrence network (genus level) using all MASH‐Ocean samples. If one genus is selected by a user, those who interact with the selected genus will be highlighted in the network. (E) Running time of the diversity analysis, function analysis, and biogeography analysis with different modes. A sample of the test, along with 150 samples of MASH‐Ocean, is used for testing different modes of function analysis. Additionally, 2147 samples are used in the test of biogeography analysis.

MASH‐Ocean also provides a comprehensive diversity analysis using taxonomic information produced through the annotation of metagenomic reads. Users can either generate microbial diversity profiles at different taxonomic levels or statistically compare the diversity among different samples by setting up distinct groups. In general, this platform allows users to establish five groups in total to carry out the diversity comparison, with at least three selected samples in each group. The diversity statistical comparison results can be divided into different taxonomic levels, that is, archaea, bacteria, eukaryota, and viruses, each of which contains six ranks including Phylum, Class, Order, Family, Genus, and Species. In total, four types of comparisons are included in the platform (Figure 2A). The first diagram is the taxonomic composition of the selected samples calculated by Kraken2 and Bracken programs. The second analysis is the ranking of taxonomically distinctive members between different groups, which is calculated by linear discriminant analysis (LDA) effect size (LEfSe). The third analysis is the heatmap of beta‐diversity distances using Jaccard or Bray‐Curtis matrix, and a principal coordinates analysis of all selected samples will also be calculated and displayed by using the beta‐diversity distances.

### Metabolic functional pathway analysis

The function analysis is designed for the visualization of the presence and abundance of key genes and modules from the major carbon, nitrogen, and sulfur metabolic functional pathways of selected samples. These pathways are critical for global biogeochemical cycling and may therefore influence the Earth's climate. The function analysis module allows users to integrate their own data set with the MASH‐Ocean database through three options. One can upload a file with the Kyoto Encyclopedia of Genes and Genomes (KEGG) Orthology (KO) list with the sequencing depth corresponding to those KOs. Users can also upload a file with the predicted ORFs ID and annotated KOs, as well as a sequencing depth file together. The final result will be displayed as a KEGG map, and the relative abundance of metabolic functional genes are shown in red gradient color (Figure 2B). If the number of uploaded or selected samples exceeds three, the relative abundance of metabolic functional genes and further distilled KEGG modules will be displayed in a heatmap for better exhibition.

### Biogeography analysis

MASH‐Ocean contains the function of interactive biogeography visualization and analysis. It supports global distribution display for both microbial species and metabolic functional genes. For microbial species analysis, users can either enter a species name or upload a genome to check their global distribution pattern in all or selected metagenomic data set in MASH‐Ocean (Figure 2C). MASH‐Ocean also allows users to upload their genomes and will be classified using GTDB‐Tk version 2.3.2 [18] for taxonomic assignment. Similarly, for metabolic functional genes, one can select the gene name provided within the website or enter a KO entry and then it will be displayed on the global map on our website.

### Interaction network analysis

MASH‐Ocean also contains the function of interactive network visualization. Six pre‐established global microbial co‐occurrence networks on the species, genus, family, order, class, and phylum levels were integrated. If users focus on the interaction of one or several specific microbes in the global oceanic microbial network, they can enter the name or taxonomic ranks of the microbe. Then this microbe will be highlighted in the entire network and users can either download the complete network or the network of their focused microbe (Figure 2D). The co‐occurrence networks were generated using FastSpar v1.0.0 [19] and iDIRECT [20], and parameters are listed in Table S2.

### Estimation for running time of diversity, function, and biogeography analyses

MASH‐Ocean takes approximately 10–50 min to process the diversity analysis module (Figure 2E), depending on the scale of analysis (ranging from 6 to 150 samples). For the function analysis and biogeography module, the time requirements vary due to the time‐consuming sequence preprocessing steps. For instance, when annotating metabolic functions in the function analysis module, MASH‐Ocean requires around 1 h for one file with size ~50 MB. In contrast, other modes complete this task in less than 5 min. For the biogeography module, MASH‐Ocean processes one genome file in approximately 35 min, and a protein sequence file in about 10 min, while other modes complete tasks with similar sample scales in less than 20 s.

### Future perspective

As the initiative platform for the MASH project in China, MASH‐Ocean offers a convenient and stable service to ensure the integration of omics data generated from hydrosphere ecosystems. In comparison to existing omics online servers, such as IMG/M [8], which focused mainly on storage of metagenomic data with limited capabilities in systematic comparison and visualization, while Wekemo Bioincloud [10] or MicrobiomeAnalyst [11] that specifically provided online analysis tools for meta‐omics, MASH‐Ocean is designed to provide exploratory insights from a big data perspective for researchers through a one‐station service. In the future, besides ocean ecosystems, MASH will include other hydrosphere ecosystems such as wetlands, lakes, rivers, and so on. For the MASH‐Ocean platform, there are three aspects that will be upgraded in the coming years. First, the data collection process will continue to accommodate more publicly available resources. Second, except for metagenome, we will include published metatranscriptome data in the future version. Third, we also aim to include microbial genomic data to enhance the integrity of MASH‐Ocean. Last but not the least, we will further closely cooperate with other databases and online analysis platforms such as the integrated microbiome analysis cloud platform, an eLibrary of Microbial Systematics and Genomics, NODE databases to enlarge and diversify the content of the MASH‐Ocean platform. Additional suggestions and comments are also welcome from our readers and users.

## AUTHOR CONTRIBUTIONS

Yinzhao Wang conceived and designed the platform. Liuyang Li, Qiang Li, Yaoxun Hu, Wenjie Li, and Zhile Wu developed the scripts and web server. Yinzhao Wang and Liuyang Li wrote the manuscript. Guoping Zhao, Fengping Wang, and Guoqing Zhang supervised this project and revised the manuscript. All authors have tested the web server, suggested amendments, read the final manuscript, and approved it for publication.

## CONFLICT OF INTEREST STATEMENT

The authors declare no conflict of interest.

## ETHICS STATEMENT

No animals or humans were involved in this study.

## Supporting information


**Movie S1**. The guidance video for operation of the MASH‐Ocean platform.

**Table S1**. Preset keywords to collect metagenomes from different types of marine habitats.
**Table S2**. Parameters to generate co‐occurrence networks.

## Data Availability

Data sharing is not applicable to this article as no datasets were generated or analyzed during the current study. This manuscript does not generate any code or data. Supplementary materials (tables, graphical abstract, slides, videos, Chinese translated version, and update materials) can be found in the online DOI or iMeta Science http://www.imeta.science/.
